# Gender Disparities in Adverse Events Resulting From Low-Value Practices in Family Practice in Spain: A Retrospective Cohort Study

**DOI:** 10.3389/ijph.2024.1607030

**Published:** 2024-07-16

**Authors:** José Joaquín Mira, Concepción Carratala-Munuera, María Asunción Vicente, Maria Pilar Astier-Peña, Daniel García-Torres, Cristina Soriano, Alicia Sánchez-García, Elisa Chilet-Rosell, Vicente F. Gil-Guillén, Adriana López-Pineda, Virtudes Pérez-Jover

**Affiliations:** ^1^ Alicante-Sant Joan Health District, Alicante, Spain; ^2^ ATENEA Research, Foundation for the Promotion of Health and Biomedical Research of Valencia Region (FISABIO), San Juan de Alicante, Spain; ^3^ Health Psychology Department, Miguel Hernandez University of Elche, Elche, Spain; ^4^ RICAPPS—Red de Investigación en Cronicidad, Atención Primaria y Prevención y Promoción de la Salud, Sant Joan Alacant, Spain; ^5^ Clinical Medicine Department, Miguel Hernandez University of Elche, Elche, Spain; ^6^ Universitas Health Centre, Zaragoza, Spain; ^7^ Public Health Service of Aragon, University of Zaragoza, Grupo de Investigación en Bioética de Aragón, Instituto de Investigación Sanitaria de Aragón, Aragón, Spain; ^8^ Centro de Salud de Mutxamel, Mutxamel, Spain; ^9^ Public Health, History of Medicine and Gynecology Department, Miguel Hernandez University of Elche, Elche, Spain; ^10^ Calitè Research, Applied Psychology Research Centre, Miguel Hernández University, Elche, Spain; ^11^ Hospital Psychology Research Group, Instituto de Investigación Sanitaria y Biomédica de Alicante (ISABIAL), Hospital General Universitario de Alicante, Alicante, Spain

**Keywords:** overuse, low value practice, adverse events, patient safety, primary care

## Abstract

**Objectives:** This study aimed to evaluate gender-based disparities in preventable adverse events due to low-value practices (LVPs) in primary care.

**Methods:** A retrospective cohort study in Alicante, Spain.

**Results:** A total of 1,516 patient records were examined, finding that older individuals and women experienced more LVP-related events. Female patients faced a higher volume of such events than males with the same health issue. Interaction analysis revealed patients treated by male physicians had more severe events, while those attended by females experienced milder ones. Adverse events were more frequent in LVPs associated with gender-based reasons.

**Conclusion:** These results highlight the need for tailored healthcare professional awareness programs on overuse’s impact on safety. Addressing outcome differences between male and female patients should inform awareness campaigns.

## Introduction

Despite the rising costs in developed Western societies, patient outcomes remain suboptimal [1], and adverse events continue to pose a significant challenge across all healthcare systems [2–4]. Due to its role in orchestrating patient flow within the healthcare system, primary care is pivotal in achieving favorable patient outcomes [5]. Although less studied, one of the causes of adverse events in primary care is directly related to recommending, administering, or prescribing healthcare services that are unlikely to benefit patients [6–8], which we consider as overuse [9].

The volume of patients subjected to low-value practices (LVPs) in the United States, Canada, Australia, and Sweden reach up to 80%, depending on the type of practice [10]. In Primary Care in Spain [11], nearly 6 out of 10 adult patients and 4 out of 10 pediatric patients annually receive at least one prescription classified as overuse. Examining only the overage in expenses resulting from unnecessary prescriptions of benzodiazepines, NSAIDs, lipid-lowering agents, paracetamol, and ibuprofen within a single year, reveals an annually total surpassing 290 million euros. This constitutes 2.8% of the entire Spanish pharmaceutical expenditure in 2018 [12], accounting solely for the cost of the prescribed medications. The continued occurrence of overuse in primary care is frequently linked to various factors, including limited time, constrained access to comprehensive patient data, defensive medical practices, and the approval of prescription decisions either made by healthcare colleagues or requested by patients [7, 13–15].

Recent studies also highlight differences in the frequency of LVPs between male and female patients [16]. Moreover, the number of adverse events due to overuse has been suggested higher in women [8]. Although it is known that women are negatively affected by a gender bias in the therapeutic effort, and they experience greater delays in diagnosis [17–19], the male and female difference has not yet been investigated in relation to overuse, which means that interventions aimed at reducing it do not consider the differential impact on female patients, who could be particularly and negatively affected by its consequences. Therefore, the overarching aim of this research is to assess if there are differences among male and female patients treated by male or female family physicians with regard the occurrence of preventable adverse events due to LVPs in the primary care setting.

In this study, we reach out to test the following hypotheses developed based on the results of previous studies within primary care [8, 20, 21].

H_1_. A higher number of LVPs are identified among female patients compared to male patients within similar age groups and reasons for consultation.
H_2_. Male and female family physicians are responsible of a similar number of LVPs among their patients.
H_3_. A higher number of preventable adverse events related to LVPs are identified among female patients compared to male patients within similar age groups and reasons for consultation.
H_4_. Male and female family physicians are involved in a similar number of preventable adverse events related to LVPs among their patients.
H_5_. Preventable adverse events stemming from overuse, either due to conditions or symptoms more commonly found in patients of a specific sex or those attributed to gender-related reasons, exhibit similarity.

## Methods

### Design

A retrospective cohort study in which a random selection of patients attending primary care consultations in Alicante province (Spain) was performed. The STROBE checklist was used as a guide for reporting the study [22]. The study protocol was published first [23].

### Primary Care in Spain

Spanish primary care is a cornerstone of the country’s healthcare system, offering accessible, and comprehensive healthcare services to individuals who require ongoing medical attention, often due to chronic illnesses. This level of care ensures universal access to quality healthcare for individuals of all ages. Preventive care, early intervention, and continuity of care is provided by multidisciplinary teams of family physicians, pediatricians, nurses, and allied health professionals.

### Definitions

In this study, overuse was defined as continuing to do what should not be done (e.g., ignoring the “Do Not Do” recommendations). The LVPs considered in the study were derived from the Spanish Commitment to Quality initiative list of recommendations [24, 25], formulated according to the Choosing Wisely campaign’s methodology to mitigate overuse [26–28]. Adverse event was defined as injury resulting from medical management or a complication, rather than the underlying disease, leading to extended hospitalization and/or disability at discharge from medical care [27]. Gender bias in health refers to unjustifiable differences in treatment between women and men based on scientific evidence. This bias arises from assuming gender differences where there are none or ignoring genuine differences that necessitate a distinct approach according to evidence [28].

### Ethics

The Research Ethic Board of the Sant Joan Hospital approved the study protocol reference 21/061. It was registered on ClinicalTrials.gov https://clinicaltrials.gov/study/NCT05233852 (NCT05233852).

### Procedure

A group of reviewers (n = 40) was formed and trained in the study LVPs identification and data collection procedures. Training was provided using anonymized records. During the training, all reviewers assessed the same cases, and concordance was measured using Cohen’s kappa coefficient. A score of 0.63 or higher was deemed acceptable, while a score of 0.84 or higher was considered excellent. Training concluded once an excellent level of concordance was reached. The list of reviewers involved in this study is provided in Supplementary Material S1.

Each reviewer independently assessed selected medical records and recorded study data. Upon identifying an LVP, the reviewer evaluated potential adverse events and, if present, assessed severity and harm extent using the Woods et al [29] scale, where higher scores indicate greater severity and a stronger relationship between the practice and harm. Events with scores above 3 were classified as adverse events, while those above 4 were attributed to LVPs. A blinded recording system was employed.

### Data Collection

Data were extracted from the primary care electronic medical records database, Abucasis, between 15 March 2023 and 31 August 2023. In Alicante, as well as in the rest of Spain, all the information about a patient is registered in a unique electronic medical record. Data from medical records were collected using an electronic data collection platform, which incorporated a trigger tool to facilitate the identification and recording of adverse events. This tool, previously used in the SOBRINA study [8], was based on recommendations by Rosenberg et al [29]. The LVPs considered in this study were agreed in a previous study [30] (Table 1). An online consensus technique involving 33 health professionals from family medicine, cardiology, intensive care, and geriatrics was conducted to reach a consensus on LVPs considering three aspects: 1) if it was still a relatively frequent LVP in primary care; 2) its frequency of application was different between men and women, with a probable association with sex or gender; and 3) if the LVP could cause a severe adverse event in the patient. Panelists marked their level of agreement/disagreement on a scale of 0 (strongly disagree) to 10 (strongly agree). The resulting score was the sum of the three scales. The LVPs that yielded a score of 20 points, or more were retained (consensus criterion) and those scoring under 10 points discarded. Then, a select group of panelists were asked to review the final list of LVPs. Additionally, during a session with experts (clinicians and gender bias in health researchers), there was a debate and consensus reached on whether the differences between men and women that could be observed in these previously selected LVPs should be attributed to the presence of gender inequalities in healthcare. In cases where treatment (or test) is indicated for a condition or symptoms that are more prevalent in patients of a specific sex, it was assumed that the risk of overtreatment (or overuse) in patients of that particular sex is higher than in the other. However, when there is no evidence that the symptoms or prevalence of the condition for which the treatment is provided differ between sex, it was assumed that differences in practice application are due to gender-related reasons. We used a scale ranging from −5, entirely attributable to belief on that are more prevalent in patients of a specific sex, to +5, entirely attributable to gender bias. Table 1 shows the outcome of this consensus among experts.

**TABLE 1:** Low value practices considered in this study (Gender Bias in Overuse in Primary Care, Spain, 2023).

Low value practice definition	Sex/Gender^a^
Benzodiazepines with long half-life (ATC codes: N05B, N05BA01, N05BA02, N05BA03, N05BA05, N05BA09, N05BA11, N05BA14, N05BA17) administered for the treatment of insomnia (ICD-10-ES codes: G47.0, F51.0, F51.01, F51.02, F51.03, F51.04, F51.05, F51.09, F51.8, F51.9, F51.19) in individuals over 65 years old	+3
Using benzodiazepines (ATC codes: N05B, N05BA01, N05BA02, N05BA03, N05BA05, N05BA09, N05BA11, N05BA14, N05BA17) for the treatment of agitation (ICD-10: R45.1) or delirium (ICD-10: R41.0, R41.82, F.22, F.43, F.05, F03.90,1) in elderly individuals (60 years or older)	+3
In patients with difficulty maintaining sleep (ICD-10: G47), using hypnotics (ATC codes: N05C) without having a previous etiological diagnosis	+4
Using opioids (ATC codes: N02A) as symptomatic treatment for primary headache (ICD-10: R51, R51, G44.51, G44.52, G44.53, G44.59, G44.81, G44.82, G44.83, G44.84, G44.85, G44.89, G44.0, and G44.2)	+2
Prescribing opioids (ATC codes: N02A) for acute disabling low back pain (ICD-10: M54.4-, M54.5) before evaluating and considering other alternatives	+2
Using non-steroidal anti-inflammatory drugs (NSAIDs) (ATC codes: M01A, M01AA, M01AB, M01AC, M01AE, M01AG, M01AH, M01AX, M01BA) in individuals with hypertension (ICD-10: I10, I11, I11.0, I11.9, I12, I12.0, I12.9, I13, I13.0, I13.1, I13.2, I13.9, I15, I15.0, I15.1, I15.2, I15.8, I15.9), heart failure (ICD-10: I50, I13.0, and I13.2), or chronic kidney disease (ICD-10: N18, I12, I12.0, I12.9, I13, I13.0, I13.1, I13.2, I13.9, I13.10, I13.11, I13.13) from any cause, including diabetes	−4
Utilizing two or more non-steroidal anti-inflammatory drugs (NSAIDs) (ATC codes: M01A, M01AA, M01AB, M01AC, M01AE, M01AG, M01AH, M01AX, M01BA) concurrently	−3
Prescribing proton pump inhibitors (PPIs) (ATC codes: A02B) as gastroprotection in patients without risk factors for gastrointestinal complications (ICD-10: K29, K22.1)	−3
Using acetylsalicylic acid for primary prevention in individuals without cardiovascular disease with treatment lasting more than 12 weeks	+2
Performing imaging tests (X-ray, MRI, CT scan) in patients with acute low back pain without red flags or indicating a CT or MRI scan in nonspecific cervical or lumbar pain without red flags	+2
Suggesting bed rest for patients with acute or subacute low back pain	+3
Ordering a CT scan or an MRI for non-specific cervicalgia or lumbago without alarm signs	+2
Prescribing analgesics (NSAIDs, paracetamol, and others) (ATC codes: M01A, M01AA, M01AB, M01AC, M01AE, M01AG, M01AH, M01AX, M01BA, N02BE01) for more than 15 days per month in a primary headache (ICD-10: R51, G44.51, G44.52, G44.53, G44.59, G44.81, G44.82, G44.83, G44.84, G44.85, G44.89, G44.0, and G44.2) that does not respond to treatment	+3
Prescribing treatment (ATC codes: G04BD) for overactive bladder (ICD-10: N39.41 and N32.81) without excluding other pathologies that may cause similar symptoms	+3
Using antipsychotics (ATC codes: N05A) for the treatment of Generalized Anxiety Disorder (ICD-10: F41.1, F41.0, F41.8, F41, F41.3, and F41.9) in Primary Care	+4
Prescription of a new drug for a previous diagnosis without a new patient assessment	+2

^a^ Results of the scale ranging from −5, entirely attributable to belief on that the condition is more prevalent in patients of a specific sex, to +5, entirely attributable to gender bias.

### Sample

The proportion of medical records with at least one LVP was expected to be 50% [8]. With an alpha risk of 0.05 and an accuracy of 2.5%, the minimum required sample size was determined to be 1,538 medical records (50% of which were from women). The study sample was stratified by age group and sex, considering the visit frequencies recorded in the National Health System’s primary care information system for 2018. Study participants were divided into three age groups: 18–59 years, 60–74 years, and >75 years, based on reference ages from prior studies [30]. A simple random sampling method with k = 5 was used to select the medical records of patients attended in the past 3 years.

### Data Analysis

Considering the higher frequency of female patients attending primary care consultations [31, 32] (In Spain, 9.6 vs. 5.7 visits per year in 2022 [33]) the adjusted LVPs and preventable adverse events rates have been calculated to correct for this effect in the interpretation of the data. The chi-square test with Yates correction were used to compare the frequency of LVPs in men and women, and the Cochran-Mantel-Haenszel test to analyze differences in the adjusted rate between the sexes. To analyze the relationship between the presence of an adverse event (dependent variable) and the corresponding independent variables such as age, the number of daily medications, patient’s gender, physician’s gender, and their interaction, a Generalized Linear Mixed Model (GLMM) was used. This model accounts for random effects to cover cases where the same patient is affected by more than one adverse event. Statistical significance for all tests was determined at *p* < 0.05 (two-tailed). The analyses were conducted using the SPSS statistical software and the RStudio V.1.1.463 programming language.

## Results

In total, 1,538 electronic medical records were reviewed, but after exclusions (due to missing data), a total of 1,516 patients were included, being 911/1,516 (60.1%) female (Table 2). The mean age of patients attended during the study period was for male 56.4 years (SD 19.4) and female patients 55.2 years old (DT 20.8). They were taking an average of 3.7 medications daily (range 1–25). A total of 245 (68.1%) patients treated by male family physicians were taking less than five drugs per day, while 115 (31.9%) were taking five or more drugs daily. In the case of patients treated by female family physicians, 769 (67.85%) were taking less than five drugs per day and 365 (32.1%) were taking five or more drugs per day. The most frequent main diagnoses in this sample were hypertension, and Type 2 Diabetes.

**TABLE 2:** Descriptive characteristics of the sample (N = 1,516) (Gender Bias in Overuse in Primary Care, Spain, 2023).

Variable	N (%)
Male	605 (39.9)
Female	911 (60.1)
Age (mean, SD)	55.6 (SD 20.3)
Hypertension	353 (23.3)
Type 2 Diabetes	183 (12.1)
Hypercholesterolemia	83 (5.5)
Anxiety	78 (5.1)
Dyslipidemia	70 (4.6)
Patients treated by male family doctors	360 (23.7)
Patients treated by female family doctors	1,134 (74.8)
No information provided^a^	22 (1.5)
Average medications daily (mean, SD)	3.7 (4.0)
Male Family doctors	
Male patients <5 drugs daily	116 (71.6)
Male patients ≥5 drugs daily	46 (28.4)
Female patients <5 drugs daily	129 (65.1)
Female patients ≥5 drugs daily	69 (34.9)
Female Family doctors	
Male patients <5 drugs daily	292 (67.1)
Male patients ≥5 drugs daily	143 (32.9)
Female patients <5 drugs daily	477 (68.2)
Female patients ≥5 drugs daily	222 (31.8)

^a^ Information not available.

H_1_
A higher number of LVPs are identified among female patients.

The prevalence of patients suffering LVPs was 465/1,516, 30.7%. A total of 221/605 (36.5%) LVPs occurred in male patients, meanwhile 417/911 (45.7%) LVPs occurred in female patients (*p*-value = 0.022). As the patient’s age increased, the frequency of LVPs also increased (*p*-value = 0.024). The number of patients who experienced at least one LVP was 465 (male patients 170/605, 28.1% and female patients 295/911, 32.4%). Among 286 patients, two or more LVPs were registered (103/605, 17.0% male patients; 183/911, 20.1% female patients).

The data confirm H_1_, with the LVPs considered in this study being more frequent among women than among male patients.

H_2_
Male and female family physicians are responsible of a similar number of LVPs.

A total of 156/360 (43.3%) LVPs were observed in patients treated by male physicians and 482/1,134 (42.5%) in patients treated by female physicians (*p*-value = 0.950). Analyzing these LVPs considering both the patient’s sex and the professional’s sex (Table 3), it was observed that only when the family doctor was female, female patients experienced more LVPs than male patients.

The findings suggest rejecting, at least partially, H_2_, as there was a higher frequency of LVPs among female patients treated by female family physicians compared to male patients treated by the same female family physicians.

**TABLE 3:** Frequency of low value practices between male and female patients being treated by male and female family physicians (Gender Bias in Overuse in Primary Care, Spain, 2023).

	Male family physician (N = 360)			Female family physician (N = 1,134)		
	Male patients (N = 162)	Female patients (N = 198)		Male patients (N = 435)	Female patients (N = 699)	
	N (%)	N (%)	*p*-value	N (%)	N (%)	*p*-value
LVPs	64 (39.5)	92 (46.5)	0.200	157 (36.1)	325 (46.5)	<0.000

LVP, low-value-practice.

H_3_
A higher number of preventable adverse events related to LVPs are identified among female patients.

During the review of electronic medical records, a total of 124 adverse events were identified among 105 patients subjected to one or multiple LVPs in the study (124/638, 19.4%), of which 35/221 (15.8%) were experienced by male patients and 89/417 (21.3%) by female patients.

A total of 26 (26/105, 24.7%) patients experienced two or more preventable adverse events related to the included LVPs in the study. These occurrences of experiencing more than one adverse event related to LVPs were concentrated in individuals aged 60 or older. Among male patients, six (19.35%) of them experienced more than one adverse event, all of whom were treated by male physicians. Among female patients, 20 (27.03%) of them experienced more than one adverse event, of which 6 were treated by male physicians (30%) and 14 by female physicians (70%) (*p*-value = 0.465). The severity tendency of the adverse events was slightly higher in the case of female patients, but the difference was not statistically significant (*p*-value = 0.058).

The data allow us to accept H_3_ because the data trend suggests that female patients experience a higher volume of preventable adverse events related to LVPs than males treated for the same health issue.

H_4_
Male and female family physicians are involved in a similar number of preventable adverse events related to LVPs.

When analyzing the interaction between patient sex and physician sex a higher proportion of patients attended by male physicians experienced an adverse event compared to those attended by female physicians (*p*-value<0.000), and in cases where a female physicians attended, female patients experienced more adverse events than male patients (*p*-value<0.002) (Table 4). The severity of adverse events suffered by male and female patients were higher when the patients were attended by male family physicians (*p*-value<0.000). Most adverse events were related to medication (99, 79.8%). No differences were identified in the nature of the adverse events suffered by patients when treated by male and female family physicians (*p*-value = 0.286).

As the patient’s age and the number of daily medications taken by the patient increase, the number of adverse events tends to rise. An interaction effect was observed between the patient’s sex and the family physician’s sex, such that when both the physician and the patient are female, there is a significant increase in the probability of adverse events. However, when the patient is male, being attended by a female physician reduces the probability of experiencing an adverse event.

Based on suggestive data indicating that therapeutic decisions made by male or female family practice had a differentiated effect in terms of the occurrence of preventable adverse events related to LVPs among their patients of either sex, H_4_ was rejected.

**TABLE 4:** Frequency of adverse events related to low value practices in male and female patients when treated by male or female family physicians (Gender Bias in Overuse in Primary Care, Spain, 2023).

	Male family physicians (N = 156)	Female family physicians (N = 482)	
	N (%)	N (%)	*p*-value
Adverse events	50 (30.1)	74 (15.4)	<0.000
Slight harm	30 (60)	62 (83.7)	0.003
Severe harm	20 (40)	12 (16.3)	

	Male patients (N = 64)	Female patients (N = 92)		Male patients (N = 157)	Female patients (N = 325)	
	N (%)	N (%)	P-valor	N (%)	N (%)	*p*-value
Adverse events	25 (39.1)	32 (34.8)	0.706	14 (8.9)	60 (18.5)	0.002

H_5_
Overuse-related adverse events attributed to sex/gender reasons exhibit similarities in specific conditions.

Despite a similar frequency of unnecessary prescriptions or tests for both men and women, whether related to LVPs associated with conditions more prevalent in female patients or influenced by gender-based reasons, a higher number of adverse events occurred in cases linked to LVPs potentially driven by gender bias (Table 5). Consequently, H_5_ was rejected based on the data.

**TABLE 5:** Frequency of adverse events related to unnecessary prescriptions or tests associated with conditions more prevalent in female patients or influenced by gender-based reasons (Gender Bias in Overuse in Primary Care, Spain, 2023).

	Male patients		Female patients		Total		
	LVPs	AEs (%)	LVPs	AEs (%)	LVPs	AEs (%)	*p*-value (total)
It was assumed that differences in overuse are due to a more prevalent condition in specific sex than in the other	118	12 (10.2)	213	31 (14.5)	331	46 (13.9)	0.001
It was assumed that differences in overuse are due to gender-related reasons	103	23 (22.3)	204	58 (28.4)	307	81 (26.4)	

LVP, low value practice; AE, adverse event.

## Discussion

The data from this study supports the notion that overutilization poses a risk to patient safety [34]. Additionally, it suggests rejecting the assumption that the frequency of LVPs and the number of preventable adverse events involving male and female family physicians are similar; rather, it supports the idea that women experience a higher number of LVPs and related adverse events. The data suggests an interaction effect between the patient’s and physician’s gender regarding the frequency of both severe and mild adverse events, deserving further attention. This interaction may be specific to the type of LVPs studied in this research. Furthermore, LVPs influenced by gender-based conceptions are more likely to result in unsafe care.

The extent and number of LVPs and their economic impact have been studied for years in various countries and healthcare levels [10, 35, 36]. Some recent studies have emerged identifying the impact of LVPs in terms of patient safety, linking LVPs to the occurrence of preventable adverse events [37]. In one of these initial studies on this topic, our group found that female patients experienced more adverse events related to LVPs than males [8]. In this second study, we aimed to delve deeper into this issue that affects women’s health.

To address this issue, first, a set of LVPs was identified where these differences between males and females could be more pronounced. Second, a review of a set of medical records of patients of both sexes was conducted to describe the frequency at which male and female patients experienced preventable adverse events related to these LVPs.

In this study, women, whose medical records were analyzed, experienced a higher volume of these LVPs during the primary care they received. This data suggests that utilization play a significant role in overutilization. It also corroborates previous observations indicating gender differences that negatively impact the quality of care received by women [8]. This study further delves into analyzing the discrepancy in LVPs frequency between men and women, specifying that when a female patient is treated by a female physician, there is a higher likelihood (up to 7% more) of experiencing one of the LVPs analyzed in this study. These results could be due to family physicians, as suggested in other studies [38], assuming differences between men and women when there are none.

It is not new, the fact that some diseases are more often attributed to men and others to women generating a bias in diagnostic criteria and access to complementary tests or treatments [8]. However, the higher number of adverse events in those cases suspected of gender bias is a novel finding. There is evidence that shows that gender, as a social construct, has a substantial impact on health behaviors, access to and use of health systems, and health system responses [39]. Gender bias can be defined as a systematic error in the social construction of the disease’s history and symptoms, which produces inequitable responses to health problems from the health services, as well as discriminatory responses by professionals [38].

The strategies designed to reduce overutilization could consider these findings and refine their approach, recognizing that female patients have a higher probability of receiving an LVP than male patients. One possible explanation is the higher utilization or healthcare-seeking behavior among women due to a persistent gender bias in our society, where they often take responsibility for family health. Another explanation lies in the recent feminization of the medical profession, which might result in a younger female workforce and therefore, less experience among these female physicians compared to their male counterparts. It could also be attributed to patients exerting more pressure on female physicians than on male physicians to undergo diagnostic tests or specific treatments. This could be influenced by the different status assigned to female professionals, owing to the enduring gender biases [19], as opposed to their male counterparts.

Data collected reveals that nearly a quarter of LVPs ultimately result in a preventable adverse event [8]. In other words, in 2 out of every 10 LVPs, harm is caused by an action on the patient through a treatment that should not have been initiated. Similar to other studies, we have also observed that among older patients, a higher number of preventable adverse events occurred [2, 3]. In this case, the data also suggests that in more severe adverse events, the involvement of male family physicians is higher than that of female physicians. Furthermore, female patients, when treated by female family physicians, exhibited a higher proportion of mild adverse events than male patients.

We know that overutilization poses a threat to the survival of healthcare systems. Moreover, its risk to patients is becoming increasingly evident. In the majority of preventable adverse events identified, the severity of the damage was mild. However, nearly two out of ten resulted in severe permanent consequences for the patient. Both in hospitals and primary care, it has been emphasized that LVPs were not as innocuous as previously thought. Indicating, for example, a test when it’s unnecessary opens up possibilities of initiating equally unnecessary treatments, risking the patient and burdening the healthcare system with unnecessary costs, to the detriment of other patients in need of care.

Considering the latest data indicating that around 7% of patients in primary care in Spain experience an adverse event in a year, the findings of this study clearly point to overutilization as a risk factor, given that the frequency of adverse events associated with LVPs is nearly 3 times higher than expected. Other studies conducted in various countries suggest rates of adverse events in primary care ranging between 1% and 24% [40], with the most common frequency being around 6%–7%, and 1.6% considered as severe events [2].

LVPs pose a threat to the sustainability of healthcare systems due to the increased costs they entail [41–43]. Initiatives implemented to reduce overuse have yielded diverse outcomes [44, 45]. The debate on overutilization and its impact on individuals and systems has expanded, verifying that multicomponent interventions are the most effective in reducing overuse. These interventions, combining various elements, should incorporate information regarding biases based on sex/gender related belief that contribute to women receiving more LVPs, especially when some culminate in adverse events.

### Implications

These findings have implications for the content of programs aimed at raising awareness among professionals about the impact of overuse on health outcomes. Given these data, it is advisable to address these potential differences in outcomes between male and female patients when planning awareness campaigns. Some examples highlight that collaboration between patients, caregivers, and clinicians yields positive outcomes in primary care, and a similar approach could be pursued in this case to reduce overuse and concurrently enhance patient safety [46]. Decision aids aimed at increasing patient safety could consider these results to prioritize situations where differences between men and women are more pronounced. Moreover, in clinical practice, particularly concerning these LVPs, clinicians should consider that an unnecessary indication may have an unexpected and negative impact leading to adverse events. Therefore, when making decisions, they should acknowledge that a low-value indication is not harmless and may negatively affect patient safety. They should assess whether the therapeutic approach is disproportionately affecting female patients compared to male patients, inadvertently impacting their health status. Finally, patient schools (e.g., patient experts) and informal caregiver education could serve as suitable platforms to educate both groups about the risks of LVPS in terms of patient safety. In essence, as patient safety remains a challenge for all primary care professionals [47], this data suggests initiating discussions about how overuse compromises patient safety. Despite practices that may seem inconsequential, they can result in a suboptimal level of care.

These results raise new questions. For instance, to what extent do defensive medicine practices causing overuse differ between male and female professionals, and which patient profiles are more susceptible? Additionally, do decision aids integrated into digital systems reduce disparities in LVPs between male and female patients? Studies on overutilization have identified the frequency of various LVPs in different countries. However, transnational comparisons of these LVPs have not been conducted and could be valuable in determining which strategies are more effective in reducing overuse, considering diverse factors, among male and female patients.

### Limitations

The sample size was calculated for a set of LVPs and not to determine the impact of gender on the outcome variables for each individual LVP. This study did not delve into differentiating whether the found differences were due to sex (biological) or gender (social) issues. Since the medical record system (Abucasis) does not include data on race, ethnic group, or socioeconomic status, these variables could not be considered. The clinical experience of the professionals who attended to the patients whose medical records were reviewed could not be determined since such information is not encoded and accessing it would have compromised the anonymization of the data. The data extraction for professionals was limited to gender. Professionals did not review their own histories, all coding and recording of information relied on the work of the reviewers. These data were collected from a limited number of cases of each LVP. More work is needed to understand the drivers of low-value care on males and females when attended by male and female family physicians.

### Conclusion

The prescriptions and tests considered of low value for the patient, as studied in this research, correspond to common and frequent situations in primary care. They represent a small part of the myriad of conditions addressed at this healthcare level, showcasing only a fraction of the broader reality within primary care settings. Consequently, they serve as a mere sample, underscoring a much larger reality where overuse poses a severe challenge for professionals, patients and healthcare systems. This issue not only jeopardizes patients but also poses a risk, as although the majority of safety incidents are deemed minor and lack permanent consequences, our findings indicate that in some cases, they significantly impact patients’ health. Moreover, these results prompt a deeper reflection and exploration into the influence that gender differences—stemming from both biological and social reasons—might have on overuse and the frequency and nature of associated safety incidents.
