# Supplementary material for: Gender Disparities in Adverse Events Resulting From Low-Value Practices in Family Practice in Spain: A Retrospective Cohort Study
Source: Int J Public Health. 2024 Jul 16;69:1607030. doi: 10.3389/ijph.2024.1607030 (PMC11286494; doi:10.3389/ijph.2024.1607030)
Supplement: Supplementary file 1 [file Table1.DOCX]

**Supplementary material 1**. List of reviewers involved in the review of electronic medical records.

Cristina Morant Sanz, María Blanes Marrtí, Inmaculada Candela Garcia, Verónica González Beneyto, Alex López Sayas, Andrea Hurtado Gutiérrez, Esther Gracia Soguero, Aida Huesa Perales, Raúl Candela Paya, Nieves Gomez Moreno, Veronica Torras Vives, Arnau Graset Tarrago, Esther Calderón Pérez, Fernando Nacher Ordóñez, Tania Ruiz Aguilar, Isabel Hervella
